# Supplementary figures and images for: Clinical interpretation of variants identified in RNU4ATAC, a non-coding spliceosomal gene
Source: PLoS One. 2020 Jul 6;15(7):e0235655. doi: 10.1371/journal.pone.0235655 (PMC7337319; doi:10.1371/journal.pone.0235655)

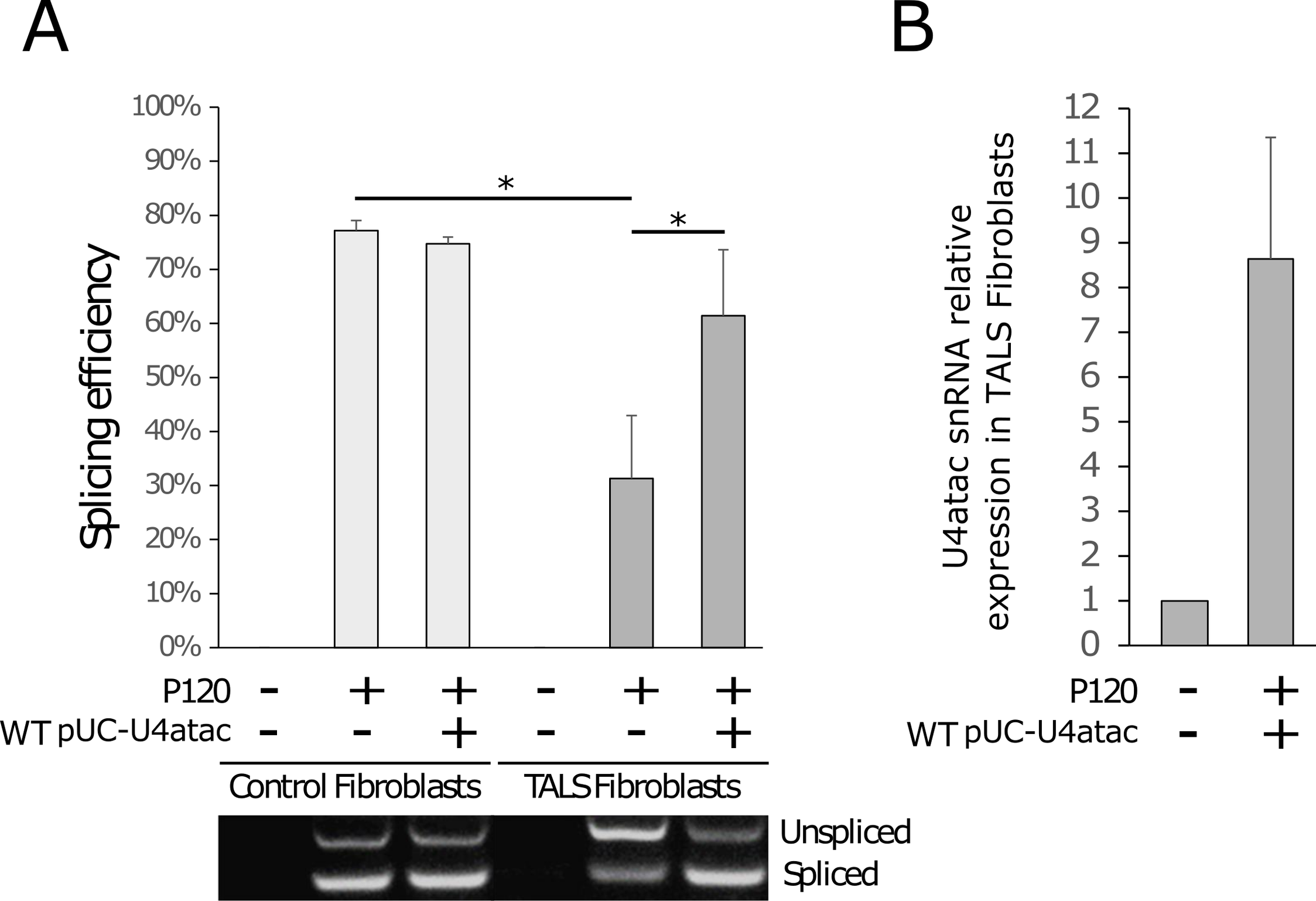

Supplement: S1 Fig — A. Splicing efficiency of the U12-type reporter intron in control or TALS fibroblasts non transfected (-) or transfected (+) with the P120 and WT pUC-U4atac plasmids, as indicated. Error-bars represent standard error of the mean (SEM) of at least three independent experiments. Statistically significant differences in splicing efficiency are indicated by an asterisk (P-values < 0.05). PCR products amplified from unspliced and spliced transcripts are shown. B. Relative amount of U4atac snRNA in TALS fibroblasts non transfected (-) or transfected (+) with the P120 and WT pUC-U4atac plasmids, as indicated. (TIF) [file pone.0235655.s001.tif]

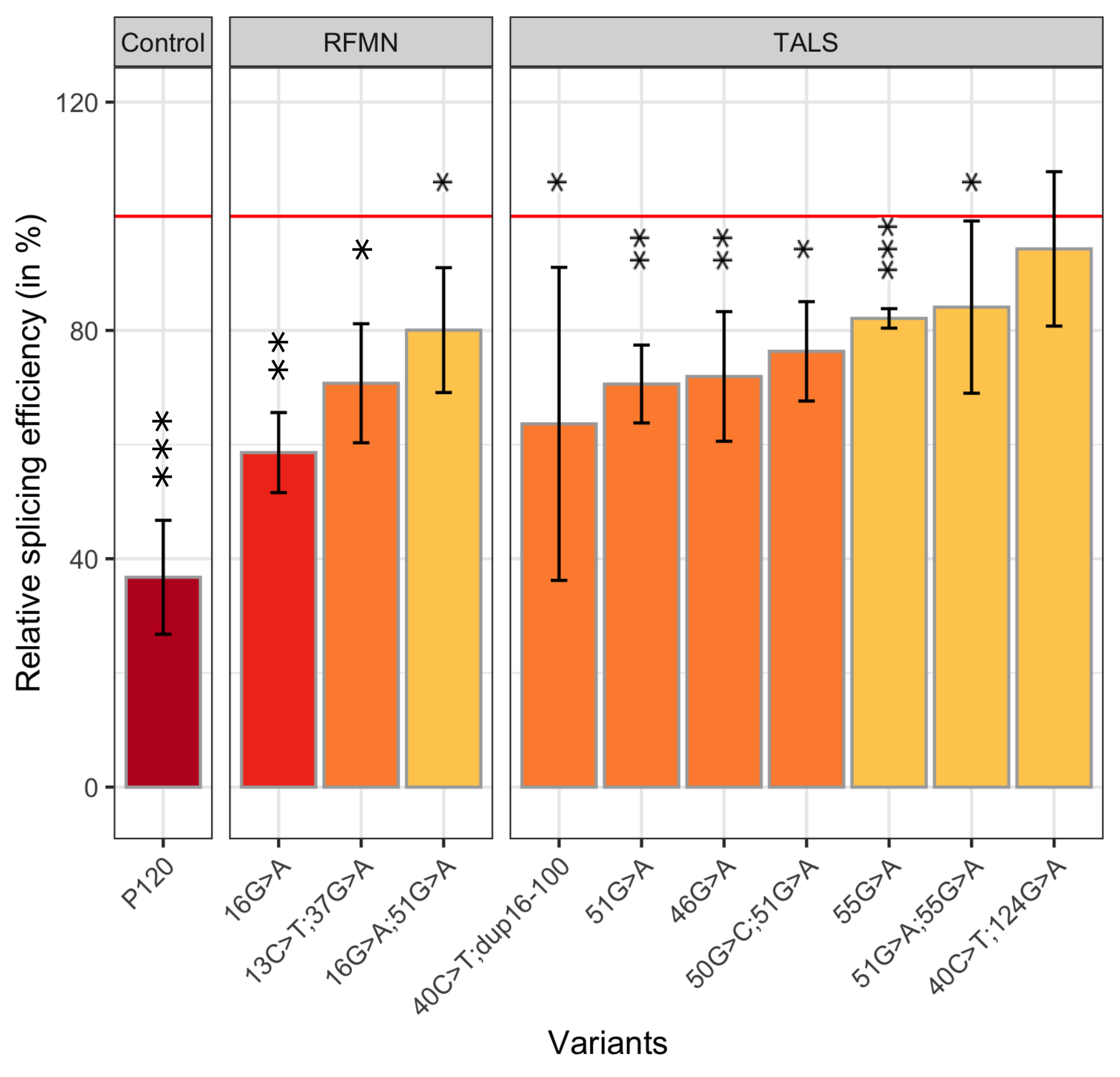

Supplement: S2 Fig — Error-bars represent standard error of the mean (SEM) of at least three independent experiments. Statistically significant differences in splicing efficiency are indicated by asterisks: * P-values < 0.05; ** P-values < 0.01 and *** P-values < 0.001 (one-tailed t-test). The red horizontal line indicates 100% splicing efficiency. (TIF) [file pone.0235655.s002.tif]
